# Disease-specific definitions of vitamin D deficiency need to be established in autoimmune and non-autoimmune chronic diseases: a retrospective comparison of three chronic diseases

**DOI:** 10.1186/ar3161

**Published:** 2010-10-14

**Authors:** Anna R Broder, Jonathan N Tobin, Chaim Putterman

**Affiliations:** 1 Division of Rheumatology, Albert Einstein College of Medicine/Montefiore Medical Center, 1300 Morris Park Avenue, Bronx, NY 10461, USA; 2 Department of Epidemiology and Population Health, Albert Einstein College of Medicine, 1300 Morris Park Avenue, Bronx, NY 10461, USA; 3 Clinical Directors Network (CDN), 5 West 37th Street, New York, NY 10018, USA

## Abstract

**Introduction:**

We compared the odds of vitamin D deficiency in three chronic diseases: systemic lupus erythematosus (SLE), rheumatoid arthritis (RA), and type 2 diabetes (T2DM), adjusting for medications, demographics, and laboratory parameters, common to all three diseases. We also designed multivariate models to determine whether different factors are associated with vitamin D deficiency in different racial/ethnic groups.

**Methods:**

We identified all patients with non-overlapping diagnoses of SLE, RA, and T2DM, with 25-hydroxyvitamin D (25OHD) levels measured between 2000 and 2009. Vitamin D deficiency was defined as 25OHD levels <20 ng/ml, based on previously established definitions. Race/ethnicity was analyzed as African-American non-Hispanic (African-American), Hispanic non-African-American (Hispanic), and Other based on self report.

**Results:**

We included 3,914 patients in the final analysis: 123 SLE, 100 RA, and 3,691 T2DM. Among African-Americans the frequency of vitamin D deficiency was 59% in SLE, 47% in RA, and 67% in T2DM. Among Hispanics the frequency of vitamin D deficiency was 67% in SLE, 50% in RA, and 59% in T2DM. Compared with the SLE group, the adjusted odds ratio of vitamin D deficiency was 1.1, 95% CI (0.62, 2.1) in the RA group, and 2.0, 95% CI (1.3, 3.1) in the T2DM group. In the multivariate analysis, older age, higher serum calcium and bisphosphonate therapy were associated with a lower odds of vitamin D deficiency in all three racial/ethnic groups: 1,330 African-American, 1,257 Hispanic, and 1,100 Other. T2DM, serum creatinine, and vitamin D supplementation were associated with vitamin D deficiency in some, but not all, racial/ethnic groups.

**Conclusions:**

Vitamin D deficiency is highly prevalent in our patients with SLE, RA, and T2DM. While the odds of vitamin D deficiency are similar in RA and SLE patients in a multivariate analysis, T2DM patients have much higher odds of being vitamin D deficient. Different demographic and laboratory factors may be associated with vitamin D deficiency within different racial/ethnic groups. Therefore, disease-specific and race/ethnicity-specific definitions of vitamin D deficiency need to be established in future studies in order to define goals of vitamin D replacement in patients with autoimmune and non-autoimmune chronic diseases.

## Introduction

Vitamin D deficiency and insufficiency, defined as 25-hydroxyvitamin D (25OHD) levels below 20 ng/ml and 30 ng/ml, respectively [1], are very common in patients with autoimmune and non-autoimmune chronic diseases, including systemic lupus erythematosus (SLE), rheumatoid arthritis (RA), and type 2 diabetes (T2DM) [2,3].

While several studies explored factors associated with vitamin D deficiency in SLE [4-9], only a few studies have compared vitamin D status in SLE and other autoimmune and non-autoimmune chronic diseases, with mixed results. Two cross-sectional studies demonstrated that SLE patients had significantly lower vitamin D levels compared with rheumatoid arthritis and osteoarthritis patients [10,11]. However, these studies included relatively small numbers of patients, and used bivariate comparisons without adjusting for medications, demographics, and laboratory parameters. In a pilot study from Canada, when vitamin D status was compared in 25 patients with SLE and a demographically similar group of 25 fibromyalgia patients, no statistically significant differences were found between the two groups, with half of the patients in both groups being vitamin D deficient [12].

Furthermore, while it is well established that African-Americans and Hispanics have lower vitamin D levels in several diseases as well as in the general population [8,13-15], only a few studies have evaluated whether different demographic and laboratory factors were associated with vitamin D deficiency in different racial/ethnic groups [16,17]. Patients with autoimmune diseases were not included in these studies. In addition, recent studies have suggested that vitamin D replacement goals may be different in Caucasians and in African-Americans with non-autoimmune diseases. While calcium and vitamin D supplementation in Caucasians is associated with a decreased risk of fractures [18], higher vitamin D levels may be associated with increased arterial plaque, a measure of cardiovascular risk, in African-Americans with diabetes [19]. This important question has not been addressed in SLE or RA, even though African-Americans and Hispanics are at a higher risk for vitamin D deficiency, and suffer more severe manifestations of SLE and RA [20].

Therefore, we designed a study to compare the odds of vitamin D deficiency in an ethnically diverse retrospective cohort with three chronic diseases: SLE, RA, and T2DM. T2DM was chosen as a non-autoimmune disease comparison group since as with SLE and RA, it is a chronic disease with wide range of manifestations, a variable course, requires multiple medications to control, and may have multi-system involvement, including renal, cardiovascular, and metabolic bone disease. Furthermore, laboratory and epidemiologic studies suggest that vitamin D deficiency may play a role in the pathogenesis of RA [20,21], SLE [22], and T2DM [23].

## Materials and methods

### Patients

We identified all patients with ICD9 (International Classification of Diseases, 9th revision) diagnoses of SLE (710.0, 695.4), RA (714.0, 714.2) and T2DM (250.*, excluding ICD9 codes for type 1 diabetes) with at least one 25-hydroxyvitamin D (25OHD) measurement occurring between January 2000 and August 2009 at Montefiore Medical Center (MMC), a large urban tertiary care center in the Bronx, NY, the University Hospital for the Albert Einstein College of Medicine. Patients were identified from the Montefiore electronic record system using Clinical Looking Glass (CLG), a software application developed at MMC, which allows clinicians and researchers to identify populations of interest, laboratory data, medications, and demographics from the MMC database [24].

Because of the retrospective nature of this study, it was not necessary to obtain informed consent from the patients, as no identifying information was stored or used in the data analysis. This project was approved by the Institutional Review Board at Albert Einstein College of Medicine/Montefiore Medical Center.

All SLE and RA diagnoses were reviewed by a rheumatologist (A.B.) who was not aware of the vitamin D status prior to reviewing the charts. It was not feasible to review all T2DM charts or to document fasting glucose measurement from the electronic database to use the American Diabetes Association (ADA) definition of T2DM [25]. Therefore, we only included patients with T2DM if they had a documented ICD9 diagnosis of type 2 diabetes, and a hemoglobin A1c >7.0% on at least two occasions. Patients with dual ICD9 diagnoses of SLE, RA or T2DM, or patients with coexisting ICD9 diagnoses for other autoimmune diseases were not included in our analysis.

Race/ethnicity was defined based on self report and was analyzed as African-American non-Hispanic (African-American), Hispanic non-African-American (Hispanic), and Other. Seasons were defined based on the degree of sun exposure into "light" (April to September) and "dark" (October to March) seasons. Vitamin D supplementation was analyzed as a yes/no variable. The most common dose of supplementation was 400-800 IU/day, and did not differ by disease.

### Vitamin D measurements

25OHD levels were measured in Montefiore Laboratory using Diasorin Liaison chemiluminescent assay [26] for all patients. We used the earliest recorded 25OHD measurement in our data analysis.

### Data analysis

Statistical analysis was performed using the STATA 10.0 software package (StataCorp, College Station, Texas, USA). We used ANOVA (or its non-parametric alternative, Kruskal-Wallis rank test) and the chi-square test to evaluate bivariate relationships between continuous and categorical variables, respectively, in the three disease groups.

Logistic regression was performed with 25OHD <20 ng/ml as the main outcome, and disease category (SLE, RA, T2DM), as the main variable of interest in the overall cohort, and within each racial/ethnic group. Variables were considered in the logistic regression either if they were shown to be significantly associated with 25OHD in previous studies, or if they were associated with disease category (*P *< 0.2) in the bivariate analysis. Logistic regression models were used to assess for interactions, to adjust for confounding, and to test for statistical assumptions using the Hosmer-Lemeshow goodness-of-fit statistic.

## Results

Based on our chart review, 100 patients with ICD9 diagnosis of RA and 123 patients with ICD9 diagnosis of SLE satisfied the American College of Rheumatology (ACR) criteria [27,28]. Patients with ICD9 of SLE (*n *= 43) and RA (*n *= 105) who did not meet the ACR criteria had heterogeneous diagnoses including fibromyalgia, mixed connective tissue disease, and other causes of inflammatory arthritis. While the median 25OHD levels were not statistically significantly different between confirmed SLE and "SLE by ICD9", the median 25OHD levels were statistically significantly different between confirmed RA and "RA by ICD9". Therefore, in order to minimize misclassification bias, only SLE and RA patients who satisfied the ACR criteria were included in the final analysis.

The distribution of RA, SLE, and T2DM patients who had 25OHD measured each year was similar within the study period. The median (Inter-Quartile Range, IQR) duration of follow-up, from the defining diagnosis to the time when 25OHD was measured, was 2.6 (0.6, 6.3) years in the SLE group, 2.7 (1.0, 7.5) years in the RA group, and 2.6 (0.8, 5.4) years in the T2DM group (*P *= 0.11).

Demographic and laboratory data for SLE, RA, and T2DM groups are provided in Table 1. Seventy-five percent of RA and 86% of SLE and T2DM patients were vitamin D deficient or insufficient. Compared with RA and T2DM, SLE patients were significantly younger, and there were more women in the SLE and RA groups. There were no statistically significant racial/ethnic differences among the three groups. The median (IQR) 25OHD levels were significantly higher in the RA group, 20.9 (16.1, 29.9) ng/ml, compared with the SLE group, 18.0 (11.3, 25.4) ng/ml, and T2DM group, 16.8 (11.1, 24.2) ng/ml (*P *< 0.0001).

**Table 1:** Demographic and laboratory data by disease

	SLE	RA	T2DM	*P*-value
	*n *= 123	*n *= 100	*n *= 3691	
Age, years Median (IQR)	34.5 (26.7, 47.7)	60.5 (52.6, 66.8)	63.3 (53.7, 71.7)	**<0.001**
Gender (% female)	109 (89)	88 (88)	2,686 (73)	**<0.001**
Race/Ethnicity (%)				
*African-American*	46 (39)	32 (34)	1,252 (36)	
*Hispanic*	39 (33)	42 (45)	1,176 (34)	0.199
*Other*	34 (29)	20 (21)	1,046 (30)	
*Unknown*	14 (12%)	16 (16%)	217 (6%)	
25OHD, ng/ml				
Median (IQR)	18.0 (11.3, 25.4)	20.9 (16.1, 29.9)	16.8 (11.1, 24.2)	**0.0001**
Season (%)				
*April-September*	61 (50)	50 (50)	1,698 (46)	0.47
Serum creatinine >1.5 mg/dl (%)	16 (13)	2(2)	855 (27)	**<0.001**
Serum calcium, mg/dl				
Median (IQR)	9.2 (8.9, 9.5)	9.5 (9.1, 9.7)	9.6 (9.2, 10)	**0.001**
Bisphosphonates use (%)	20 (16)	34 (34)	346 (9)	**<0.001**
Calcium supplementation (%)	60 (49)	59 (59)	864 (23)	**<0.001**
Prednisone use (%)	85 (69)	65 (65)	231 (6)	**<0.001**
Vitamin D supplementation (%)	57 (46)	56 (56)	867 (23)	**<0.0001**

SLE, systemic lupus erythematosus; RA, rheumatoid arthritis; T2DM, type 2 diabetes mellitus; 25OHD, 25hydroxyvitamin D; IQR, inter-quartile range.

The results of the multivariate analysis are summarized in Table 2. T2DM patients were twice as likely to be vitamin D deficient compared with SLE patients (*P *= 0.003). However, the odds ratio of vitamin D deficiency in RA was not statistically or clinically different compared with SLE (OR 1.1, *P *= 0.691). Younger age, African-American race, serum creatinine >1.5 mg/dl, and 25OHD measurement during the "dark" season (October to March) were associated with vitamin D deficiency in this model. Bisphosphonate therapy, vitamin D supplementation, and higher serum calcium were associated with increased odds of having 25OHD levels ≥ 20 ng/ml. Gender, calcium supplementation and prednisone use were not statistically significantly associated with vitamin D deficiency. Furthermore, patients with SLE or RA were 38% less likely to be vitamin D deficient compared with T2DM patients (95% CI 0.42, 0.91, *P *= 0.015).

**Table 2:** Logistic Regression model for 25OHD deficiency (*n *= 3,173)

	OR (95% CI)	*P *-value
SLE	1.0	
RA	1.1 (0.62, 2.1)	0.691
T2DM	2.0 (1.3, 3.1)	**0.003**
Age (for every decade)	0.83 (0.78, 0.88)	**<0.001**
Male gender	0.95 (0.79, 1.1)	0.58
Race/Ethnicity		
*African-American*	1.0	
*Hispanic*	0.75 (0.63, 0.90)	**0.002**
*Other*	0.70 (0.58, 0.84)	**<0.001**
October to March season	1.3 (1.1, 1.5)	**0.002**
Serum calcium, mg/dl	0.70 (0.63, 0.78)	**<0.001**
Vitamin D supplementation	0.81 (0.68, 0.96)	**0.015**
Bisphosphonate therapy	0.59 (0.47, 0.76)	**<0.001**
Serum creatinine >1.5 mg/dl	1.4 (1.1, 1.7)	**0.001**

SLE, systemic lupus erythematosus; RA, rheumatoid arthritis; T2DM, type 2 diabetes mellitus; 25OHD, 25hydroxyvitamin D; OR, odds ratio; CI, confidence interval.

Body mass index (BMI) data from the electronic medical record were only available for 35% SLE, 48% T2DM, and 29% RA patients within one year around the time when 25-OHD was measured. The median (IQR) BMI was statistically and clinically similar between RA and T2DM groups: 30.2 kg/m^2 ^(24.9, 36.3), and 30.3 kg/m^2 ^(26.1, 36.1), respectively. SLE patients had lower median (IQR) BMI, 24.1 kg/m^2 ^(20.2, 30.6), compared with RA and T2DM, *P *= 0.001. BMI over 30 kg/m^2 ^was associated with 25-OHD levels <20 ng/ml in the bivariate analysis, consistent with previous reports [7,29]. However, adding BMI into our model did not change the main conclusions of the study, and BMI did not appear to be a confounder.

Among African-Americans, the frequency of severe vitamin D deficiency (<10 ng/mL) was 24% in SLE, 13% in RA, and 26% in T2DM. Among Hispanics the frequency of severe vitamin D deficiency was 14% in SLE, 14% in RA, and 17% in T2DM. The rates of vitamin D deficiency and insufficiency varied by disease and by race/ethnicity (Table 3). In addition, there was a statistically significant interaction between disease category and race/ethnicity, suggesting that race/ethnicity was an effect modifier.

**Table 3:** 25OHD by race/ethnicity

	African-American	Hispanic	Other	*P*-value
SLE, n (column %)	46	39	34	
*<20 ng/ml*	27 (59)	26 (67)	15 (44)	
*≥20 and <30 ng/ml*	16 (35)	9 (23)	11 (32)	0.12
*≥30 ng/ml*	3 (7)	4 (10)	8 (24)	
Median 25OHD (IQR), ng/ml	17.7 (10.0, 24.2)	17.1 (11.3, 23.4)	21.4 (12.0, 28.4)	0.34
RA, n (column %)	32	42	20	
*<20 ng/ml*	15 (47)	21 (50)	6 (30)	
*≥20 and <30 ng/ml*	8 (25)	13 (31)	8 (40)	0.54
*≥30 ng/ml*	9 (28)	8 (19)	6 (30)	
Median 25OHD (IQR), ng/ml	21.5 (14.8, 31.1)	24.7 (20.1, 32.4)	25.3 (17.7, 32.8)	0.28
T2DM, n (column %)	1252	1176	1046	
*<20 ng/ml*	837 (67)	697 (59)	620 (59)	
*≥20 and <30 ng/ml*	249 (20)	308 (26)	280 (27)	**<0.0001**
*≥30 ng/ml*	166 (13)	171 (15)	146 (14)	
Median 25OHD (IQR), ng/ml	15.0 (9.7, 23.1)	17.8 (12.2, 24.7)	17.9 (12.1, 24.3)	**0.0001**

SLE, systemic lupus erythematosus; RA, rheumatoid arthritis; T2DM, type 2 diabetes mellitus; 25OHD, 25hydroxyvitamin D; IQR, inter-quartile range.

Therefore, we created separate logistic regression models by race/ethnicity, and the results are summarized in Tables 4, 5, 6. Older age, higher serum calcium, and bisphosphonate therapy were each associated with the lower odds of vitamin D deficiency in all three racial/ethnic groups. T2DM was associated with the higher odds of vitamin D deficiency in Others and in African-Americans, but not in Hispanics. Serum creatinine >1.5 mg/dl were associated with the higher odds of vitamin D deficiency in African-Americans and Hispanics, but not in Others. Vitamin D supplementation was associated with the lower odds of vitamin D deficiency only in Others.

**Table 4:** Logistic regression models predicting 25OHD deficiency in African-Americans, *n *= 1162

	OR (95% CI)	*P*-value
SLE	1.0	-
RA	1.22 (0.45, 3.27)	0.695
T2DM	2.4 (1.2, 4.7)	**0.014**
Age, for every decade	0.81 (0.74, 0.89)	**<0.001**
Male gender	1.1 (0.79, 1.5)	0.608
October - March season	1.4 (1.1, 1.8)	**0.013**
Serum calcium, mg/dl	0.76 (0.64, 0.89)	**0.001**
Vitamin D supplementation	0.84 (0.62, 1.12)	0.23
Bisphosphonate therapy	0.55 (0.33, 1.12)	**0.02**
Serum creatinine >1.5 mg/dl	1.6 (1.16, 2.2)	**0.004**

SLE, systemic lupus erythematosus; RA, rheumatoid arthritis; T2DM, type 2 diabetes mellitus; 25OHD, 25hydroxyvitamin D; OR, odds ratio; C, confidence interval.

**Table 5:** Logistic regression models predicting 25OHD deficiency in Hispanics, *n *= 1,068

	OR (95% CI)	*P*-value
SLE	1.0	-
RA	0.98 (0.37, 2.6)	0.977
T2DM	1.2 (0.59, 2.6)	0.560
Age, for every decade	0.80 (0.73, 0.88)	**<0.001**
Male gender	0.77 (0.56, 1.1)	0.112
October to March season	1.3 (0.98, 1.62)	0.077
Serum calcium, mg/dl	0.66 (0.55, 0.79)	**<0.001**
Vitamin D supplementation	0.92 (0.68, 1.25)	0.613
Bisphosphonate therapy	0.65 (0.44, 0.96)	**0.031**
Serum creatinine >1.5 mg/dl	1.4 (1.0, 1.97)	**0.048**

SLE, systemic lupus erythematosus; RA, rheumatoid arthritis; T2DM, type 2 diabetes mellitus; 25OHD, 25hydroxyvitamin D; OR, odds ratio; CI, confidence interval.

**Table 6:** Logistic regression models predicting 25OHD deficiency in Others, *n *= 943

	OR (95% CI)	*P*-value
SLE	1.0	-
RA	0.72 (0.18, 2.9)	0.636
T2DM	2.8 (1.3, 6.2)	**0.009**
Age, for every decade	0.88 (0.79, 0.98)	**0.02**
Male gender	1.0 (0.74, 1.4)	0.990
October to March season	1.2 (0.89, 1.5)	0.26
Serum calcium, mg/dl	0.64 (0.55, 0.81)	**<0.001**
Vitamin D supplementation	0.67 (0.49, 0.91)	**0.012**
Bisphosphonate therapy	0.53 (0.34, 0.83)	**0.005**
Serum creatinine >1.5 mg/dl	1.2 (0.85, 1.7)	0.383

SLE, systemic lupus erythematosus; RA, rheumatoid arthritis; T2DM, type 2 diabetes mellitus; 25OHD, 25hydroxyvitamin D; OR, odds ratio; CI, confidence interval.

## Discussion

In this study we showed that 25OHD deficiency and factors associated with it vary in autoimmune and non-autoimmune chronic diseases and in different racial/ethnic groups. Based on our literature review, this issue has not been previously addressed. In addition, our findings underscore the importance of using multivariate analysis when comparing 25OHD levels in different diseases. Furthermore, based on the results of our study, we were able to identify new directions for future studies.

### Factors associated with vitamin D deficiency in SLE, RA, and T2DM

We demonstrated that patients with T2DM have significantly higher odds of being vitamin D deficient compared with SLE and RA patients in the multivariate analysis, while the rates of vitamin D deficiency were not different between SLE and RA patients. The difference in 25OHD levels in SLE and RA compared with T2DM may reflect biases among physicians: 25OHD was measured in over 30% of all SLE and RA patients in the database compared with only 14% of T2DM patients. Moreover, T2DM patients were less likely to be prescribed calcium, bisphosphonates, or vitamin D supplementation, despite being significantly older than SLE patients. This suggests that rheumatologists, who often are both the specialty and primary care providers for SLE and RA patients, may be more aggressive at vitamin D replacement, especially since a large percentage of SLE and RA patients are on prednisone.

Alternatively, there may be disease-specific biologic mechanisms leading to the differences demonstrated in our study, such as immunity-related effects of vitamin D in SLE and RA, and metabolic effects of vitamin D in T2DM [21,30].

Another important finding of our study was that older age was associated with the lower odds of vitamin D deficiency in the overall model and across all three racial/ethnic groups. In the overall model, for every decade increase in age, the odds of 25OHD deficiency decreased by 16% (*P *< 0.001). While the mean vitamin D levels did not differ by age in the National Health and Nutrition Examination Survey (NHANES) 2001 to 2004 [13], an association between older age and higher vitamin D levels was previously reported in SLE [5]. This association has not been reported in RA or T2DM. Several behavioral factors may potentially explain this association: 1) physicians are more aware of vitamin D deficiency in older patients; 2) younger patients are more likely to use sunscreen and/or have a low intake of vitamin D rich foods, 3) older people are more likely to take vitamin D and calcium supplementation [5,31,32]. Alternatively, the association between older age and vitamin D status may be different in chronic diseases compared with the general population.

### A comparison of vitamin D levels in our patients with the previously published vitamin D levels from the NHANES 2001 to 2004 data

Although we did not have a general population serving as a control group, we were able to compare vitamin D data in our cohort with some of the vitamin D data reported by Ginde at el in African-American and Mexican-American men and women stratified by age in the NHANES 2001 to 2004 [13]. The mean/median 25OHD were comparable in all age groups in African-American men and women in our cohort and in the NHANES cohort. The rates of vitamin D <10 ng/ml were also similar. However, in our cohort the rates of vitamin D ≥ 30 ng/ml were slightly higher for African-American men and women over 40 years old compared with the NHANES cohort. 25OHD levels and the rates of 25OHD <10 ng/ml and ≥ 30 ng/ml were very different in our Hispanic patients (largely from Puerto Rico and Dominican Republic) compared with the Mexican Americans from the NHANES study, suggesting that either the groups were too heterogeneous or that different factors may be associated with the vitamin D status in different subpopulations of Hispanics.

### Limitations

Our study has several limitations, mainly related to its retrospective nature and the absence of a non-chronic disease comparison group. In order to evaluate the degree of selection bias in this retrospective analysis, we compared patients who had vitamin D measured with patients who did not have vitamin D measured within each disease category. While there were some differences with respect to the duration of follow-up, age, and laboratory parameters, these differences were independent of the disease category, and, therefore, did not significantly bias the odds ratios in our logistic regression model.

Accurate medication information was sometimes difficult to obtain retrospectively, and we did not have sufficiently detailed information about sun exposure, sunscreen use, smoking history, medication compliance, non-prescription supplementation with vitamin D, nutritional/dietary intake, use of disease modifying medications, and objective measures of disease severity. Although we believe this was a non-differential bias, medication-related information should be interpreted with caution.

Even though our study showed no association between prescribed vitamin D supplementation and vitamin D levels in African-American and Hispanics, there were both statistically and clinically significant associations between bisphosphonate therapy and vitamin D status, suggesting that patients on bisphosphonates were more likely to be taking vitamin D supplements, either by prescription or non-prescription. Careful assessment of all medication use, both prescribed and over-the-counter, is necessary to evaluate the risk of deficiency as well as total vitamin D intake.

The main objective of this study was to compare the odds of 25OHD deficiency in SLE, RA, and T2DM. Our study was not powered sufficiency to look for factors associated with vitamin D deficiency within each disease category. Therefore, we did not adjust for disease-specific factors shown to be associated with 25OHD deficiency in previous studies, such as use of Hydroxychloroquine (HCQ) in SLE [6,12], corticosteroids in SLE and RA [33], or hemoglobin A1c in T2DM [34]. According to the electronic record, 42 SLE and 6 RA patients were on HCQ at the time when 25OHD levels were measured, while none of the T2DM patients were on HCQ. In a subgroup analysis, HCQ was not significantly associated with 25OHD in our model, and did not appear to alter our main conclusions.

Although T2DM group was representative of the overall T2DM population in our medical center, these patients may have had more severe T2DM, as all of the T2DM patients in our study were treated with either insulin or oral medications for diabetes at the time when 25OHD was measured. Therefore, the conclusions reached in our analysis may not be generalizable to patients with the hemoglobin A1c ≤ 7.0% or patients with diet controlled T2DM.

Since the disease groups were independent from one another, inclusion of a disproportionately large T2DM group did not affect our ability to detect significant differences in our sample. Furthermore, when we repeated bivariate analysis and logistic regression using a random subsample of 738 (20%) T2DM patients, the main conclusions of this analysis did not change significantly.

Albumin measurements were available for only 70% of patients, and, therefore, we did not adjust our statistical model for serum albumin or for corrected calcium values. This could have affected our conclusion that low serum calcium was associated with vitamin D deficiency. In addition, 25OHD measurements themselves could be influenced by low albumin as well, since 10% to 20% of 25OHD is albumin-bound [35]. In a subgroup analysis of patients who had albumin and serum calcium measured together, both corrected calcium and albumin were statistically significant predictors of 25OHD levels. It will be necessary to address these limitations in further prospective studies.

Despite the above limitations, the prevalence of vitamin D deficiency in SLE, RA, and T2DM in our study was consistent with previous reports in SLE [8], T2DM [36], and RA [37]. In addition, in our model, the odds of vitamin D deficiency were associated with African American race, season with the lower light exposure, and elevated serum creatinine similar to previous reports [1]. Therefore, despite the retrospective nature of this analysis, our data and our conclusions are robust.

## Conclusions

Vitamin D deficiency is highly prevalent in the ethnically diverse patient population with SLE, RA, and T2DM from a large urban tertiary care center. However, the rates of vitamin D deficiency are not significantly higher than those reported nationally in the NHANES 2001 to 2004, suggesting that vitamin D deficiency in these chronic diseases reflects the overall population trends. Different demographic and laboratory factors are associated with 25OHD deficiency within different racial/ethnic groups. Moreover, the odds of 25OHD deficiency are different in T2DM compared with SLE and RA. Therefore, disease specific and race/ethnicity specific definitions of vitamin D deficiency may need to be established to determine goals of vitamin D replacement therapy, and to define outcomes in clinical studies for vitamin D repletion in autoimmune and non-autoimmune chronic diseases.

## Abbreviations

25OHD: 25hydroxyvitamin D; 95% CI: 95% confidence interval; ACR: American College of Rheumatology; ADA: American Diabetes Association; BMI: body mass index; CLG: Clinical Looking Glass; HCQ: Hydroxychloroquine; ICD9: The International Classification of Diseases, 9th revision); IQR: inter-quartile range; MMC: Montefiore Medical Center; NHANES: The National Health and Nutrition Examination Survey; OR: odds ratio; RA: rheumatoid arthritis; SLE: systemic lupus erythematosus; T2DM: type 2 diabetes mellitus.

## Competing interests

The authors declare that they have no competing interests.

## Authors' contributions

ARB and CP contributed to study design and conception, data analysis and preparation of the manuscript. ARB contributed to acquisition of data. JNT contributed to data analysis and interpretation, and manuscript preparation and revision.
